# Comparative Assessment of a Light-Curable Dental Composite Reinforced with Artificial Fibers

**DOI:** 10.3390/polym16212970

**Published:** 2024-10-23

**Authors:** Bartosz Bienias, Jolanta Kostrzewa-Janicka, Kamila Wróbel-Bednarz, Izabela Strużycka

**Affiliations:** 1 Department of Prosthodontics, Faculty of Dental Medicine, Medical University of Warsaw, Binieckiego 6 St., 02-097 Warsaw, Poland; bartosz.bienias@wum.edu.pl (B.B.); jolanta.kostrzewa-janicka@wum.edu.pl (J.K.-J.); 2 Department of Comprehensive Dentistry, Faculty of Dental Medicine, Medical University of Warsaw, Binieckiego 6 St., 02-097 Warsaw, Poland; izabela.struzycka@wum.edu.pl

**Keywords:** Fiber-Reinforced Composites (FRCs), dental composite reinforced with fibers, hybrid carbon-aramid, aramid fibers

## Abstract

FRCs (Fiber-Reinforced Composites) are materials that are being used increasingly more often in dentistry as an alternative to traditional restorations made of ceramics or metals. The aim of this study was to carry out a comparative analysis of the strength parameters of a light-curable dental composite reinforced with one single band and two single bands of artificial fibers. The specimens for the strength tests were prepared in accordance with the guidelines of the PN-EN ISO 4049:2019-07 international standard. The test material covered specimens of composite reinforced with single (one or two) bands of fibers. The following bands of fibers were used: carbon (WGL), aramid (AMD) and hybrid carbon–aramid (WGL-AMD). The presence of one single band of aramid fibers caused a three-fold increase in deflection, with a simultaneous increase in the Young’s modulus of over 140%. The flexural strength of specimens reinforced with one single band of aramid fibers was higher by 280% than that control group specimens (KONT). To summarize the performed tests, the incorporation of carbon, aramid and hybrid carbon–aramid fibers into organic matrix has a significant impact on the values of the mechanical parameters of dental composites. The results indicate that particular attention should be paid to aramid fibers, which have rarely been used in dentistry so far.

## 1. Introduction

The main directions of current research carried out for the purpose of improving the physicochemical parameters of composites focus on new types of monomers, fillers, initiators and adhesion enhancers and on introducing various artificial fibers as the reinforcement phase while at the same time reducing the weight of the composite material [1,2,3]. In FRC (Fiber-Reinforced Composite) structures, the fibers that constitute the reinforcement phase fulfill the role of improving the mechanical properties of the polymer organic matrix. The history of dental FRCs starts in the early 1960s with glass fibers and the early 1970s with carbon/graphite fibers. The first application of reinforcement was in the denture base polymer (poly(methyl methacrylate), PMMA) [4,5]. This research did not result in a clinically usable method for reinforcing denture bases and denture base polymer was again tried as a reinforcement in the late 1980s and the early 1990s with ultra-high modulus polyethylene fibers [6,7,8], glass fibers and aramid fibers [9]. Today FRCs are materials that are used increasingly more often in dentistry as an alternative to traditional restorations made of ceramics or metals [10,11,12,13]. FRCs are mainly used in direct restorative and prosthetic dental procedures, although more complex constructs are made indirectly in dental laboratories. FRCs also have potential applications in repairing old metal-based FDPs and porcelain veneer repairs [14,15], conventional removable partial and complete dentures and implant-supported overdentures [16,17,18]. Clinical experience with FRCs shows a good clinical function for restorations [19,20,21,22]. The fibers that are used most frequently in dentistry are glass fibers and, much less frequently, carbon and polyamide fibers [23,24]. Carbon fibers are composed of over 90% carbon. Their structure can be defined as a set of crystallites with a graphite structure, which have a more or less ordered layout along the fiber axis. The most desirable features of carbon fibers include small delaminations, large and developed crystallites with an orientation parallel to the fiber axis and a small defect content. Moreover, carbon fibers are characterized by high strength and a high Young’s modulus; they absorb vibrations, have a low density and a high resistance to acids and bases [25,26,27,28,29]. Aramid fibers, in turn, belong to the group of synthetic fibers that are fabricated of polyamides. Rigid aramid macroparticles are oriented parallel to the fiber axis and form planes, in which there are hydrogen bonds between the macroparticles and strong covalent bonds along the main chain, which are oriented in parallel. The high tensile strength of aramid fibers stems from the system of alternating aromatic groups and amid bonds, which form adjoining, layered, ordered structures characterized by high rigidity, a strong orientation of the macroparticles and their organization into three-dimensional spatial structures that are parallel to the fiber axis [10,11,30,31,32,33].

The aim of the study was to carry out a comparative analysis of the strength parameters of a light-curable dental composite reinforced with one single band and two single bands of artificial fibers. Furthermore, the study analyses mechanical structural changes that occur as a result of strength tests.

## 2. Material and Methods

### 2.1. Strength Tests

The specimens for the strength tests were prepared in accordance with the guidelines of the PN-EN ISO 4049:2019-07 international standard [34]. Each sample had a cuboid shape with dimensions of 25 ± 0.1 mm in length, 2 ± 0.1 mm in width and 2 ± 0.1 mm in height. The test material covered specimens of composite reinforced with single (one or two) bands of fibers. The following bands of fibers were used: carbon (WGL), aramid (AMD), and hybrid carbon–aramid (WGL-AMD) bands that were created for the purpose of this study. The Gradia Direct Posterior light-curable composite used was 7,7,9 (or 7,9,9)-trimethyl-4,13-dioxo-3,14-dioxa-5,12-diazahexadecane-1,16-diylbismethacrylate, Ytterbiumtrifluoride, (octahydro-4,7-methano-1H-indenediylbis (methylene) bismethacrylate in the A2 color/shade (GC Corporation, Tokyo, Japan) and the G Bond bonding material (GC Corporation, Tokyo, Japan) used was 2-Hydroxylethyl methacrylate, Urethanedimethacrylate, which was compatible with the composite. The composite was reinforced with bands of the aforementioned fibers. The bands were joined in the form of roving. The weight ratio of the fibers in the hybrid was 1:1. The composition and parameters of selected fibers are presented in Table 1.

The tests covered 70 specimens in total, of which 60 specimens were formed of a composite with artificial fibers used as reinforcement were the test group and 10 specimens of the composite without the addition of fibers were the control group (KONT). The test group was divided into two subgroups, each composed of 3 series of 10 specimens each. The specimens in the first subgroup were reinforced with one single band of artificial fibers (WGL 1, AMD 1, WGL-AMD 1), while the specimens in the second subgroup were reinforced with two single bands of artificial fibers (WGL 2, AMD 2, WGL-AMD 2). The specimens were subjected to strength tests in the form of the three-point flexural strength (TFS) test, which made it possible to determine the maximum bending force, deflection at failure, flexural strength and Young’s modulus. The static three-point flexural strength tests were performed with the use of a certified Zwick 1435 (Zwick/Roell GmbH & Co. KG, Ulm, Germany) tensile-testing machine with a strain gauge force measuring head sensor in the range up to 0.5 kN.

### 2.2. Microscopic Structural Tests

Following the performed strength tests, analyses of fractures and microsections of the specimens were performed with the use of the Hitachi TM 3000 (Hitachi High Technology Corporation, Tokyo, Japan) scanning electron microscope. The imaging was performed in the low vacuum mode under the pressure of 70 Pa with the use of a beam energy of 5 kV. Each specimen was observed at the following magnifications: 100×, 200×, 400×, 500× and 1000×.

## 3. Results

### 3.1. Strength Parameters 

**Figure 1:**
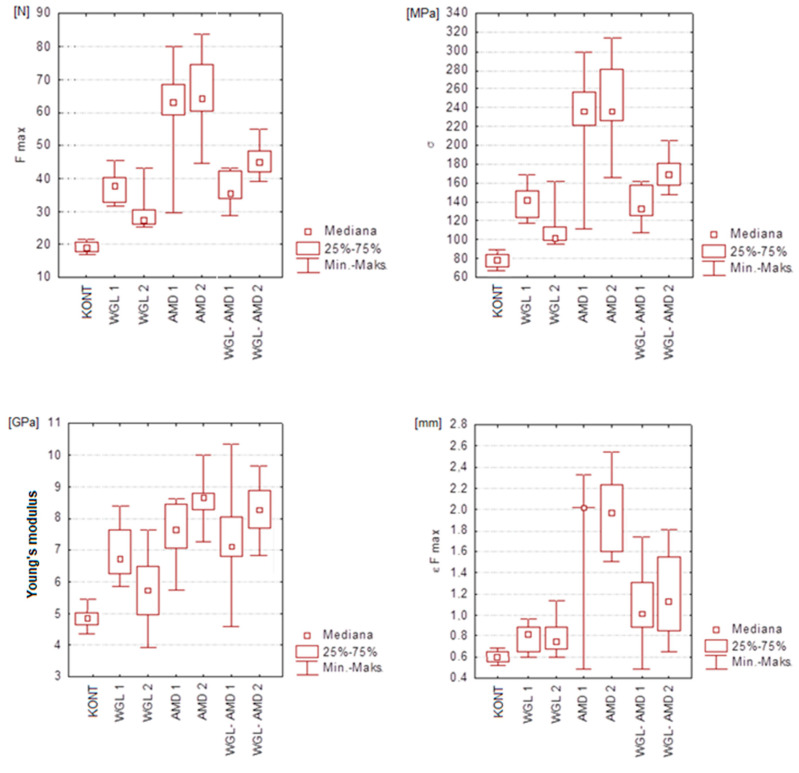
Box charts illustrating the results of analyses in the field of descriptive statistics divided into groups for the four parameters studied.

The nonparametric Mann–Whitney test was used for two independent groups and the Kruskal–Wallis test for multiple groups. The strength of the correlation of the examined strength parameters in the groups was measured by using the Pearson and Spearman correlation coefficients. The assumed significance level was α = 0.05. Among the specimens reinforced with one single band of fibers, the highest results in the four tested strength parameters were achieved by the specimens from the AMD 1 group. They achieved a maximum bending force of 63 N (*p* < 0.0001), which was three times higher than the value for the KONT group. The presence of one single band of aramid fibers caused a three-fold increase in deflection with a simultaneous increase in the Young’s modulus by over 140% (*p* < 0.0002). The flexural strength of the AMD 1 specimens was higher than that of the KONT group specimens by 280% (*p* < 0.0002) (Figure 1).

The strength tests performed proved that there were many analogies among the results achieved for the specimens reinforced with two single bands of fibers and the results achieved in the tests on the specimens with one single band of fibers. The specimens from the AMD 2 group were proved to have the highest flexural strength. The reinforcement with two single bands of aramid fibers improved the flexural strength almost three-fold and the value of the maximum bending force increased by approx. 40 N. The lowest flexural strengths with the simultaneously lowest values of the maximum bending force were achieved by the specimens from the WGL 2 group. The specimens from the AMD 2 and WGL-AMD 2 groups achieved almost twice as high values for the Young’s modulus, and thereby they proved to be the most rigid compared to the specimens from the KONT group. The Young’s moduli of these specimens exceeded 8 GPa (*p* < 0.0001), (Figure 1).

### 3.2. Observations with the Use of SEM

**Figure 2:**
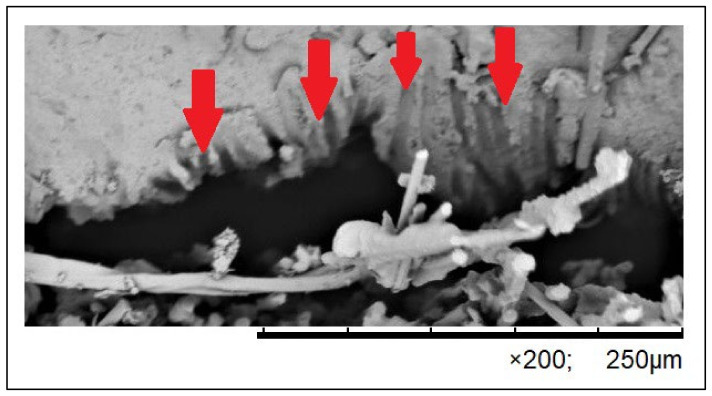
Microscopic image of the fracture of a specimen from the WGL 1 group presenting the phenomenon of debonding. Red arrows mark places where carbon fibers broke off the composite; images are presented at 200× magnification.

**Figure 3:**
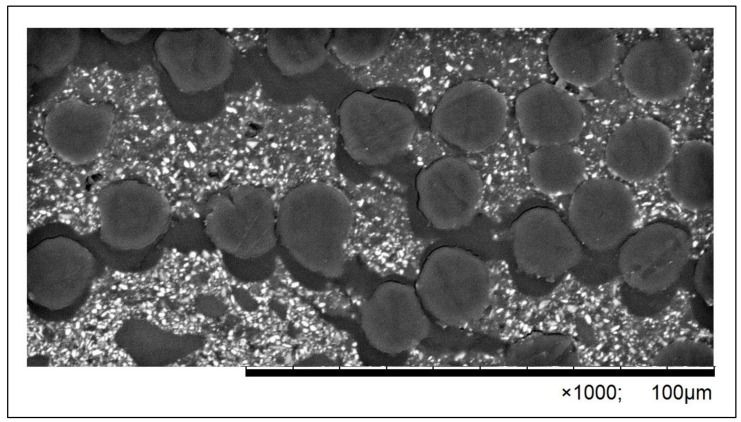
Microscopic image of the microsection of a specimen from the AMD 2 group, presenting the phenomenon of debonding inside the layer of aramid fibers; images are presented at 1000× magnification.

**Figure 4:**
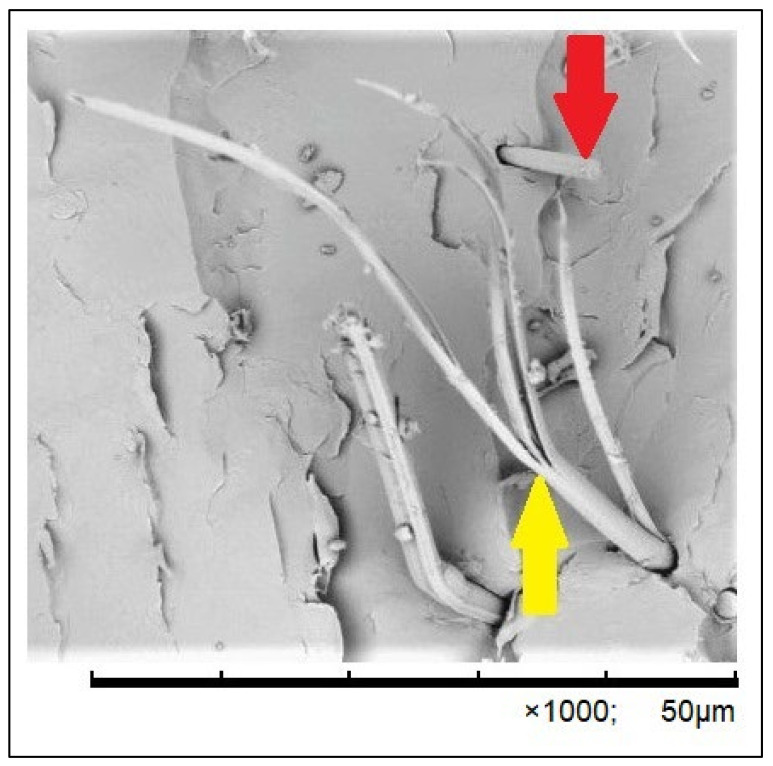
Microscopic image of the fracture of a specimen from the WGL-AMD 1 group, presenting a comparison of the ruptured single fibers: carbon fiber (marked with a red arrow) and aramid fiber (marked with a yellow arrow), at 1000× magnification.

The course of the three-point flexural strength test was recorded in the form of numerical data, serving as the basis for graphical charts illustrating the correlation between the applied force and the deformation for each specimen. The analysis of the charts with the simultaneous observation of the three-point bending process in the tested groups enabled a detailed comparative analysis of the impact of the applied force on the mechanism of specimen failure. The analyses of the samples with the use of SEM confirmed that there are many similarities between the specimens reinforced with one single band and with two single bands of fibers. The observed degradations included cracking of the fiber–composite boundary layer (debonding), cracking of the composite matrix, delaminations and failures of fibers (Figure 2, Figure 3 and Figure 4). The degradations were either local or covered a large area, encompassing many layers or the whole specimen. Various different types of degradation were frequently observed in one specimen, notably debonding, and the cracking of the fiber–composite boundary layer occurred in 90% of the specimens of the test group (Figure 2 and Figure 3). Such types of damage in the form of cracking were initiated by an accumulation of scattered microcracks and occurred in the fiber–composite boundary layer and in certain cases also inside the fiber layer. The occurrence of cracking on the fiber–composite boundary and between the fibers was caused by a loss of adhesion.

## 4. Discussion

Tests on biomaterials that are performed in laboratory conditions for the purpose of determining the optimum protocols for a clinical procedure are particularly important in the initial stages of implementation discussions [35,36,37,38]. Mechanical tests supported by a microscopic analysis provide an opportunity to gain in-depth insights into the effects that occur inside a fiber-reinforced composite material and allow for an observation of the mechanical degradation [36,37,38]. All specimens in the test group which contained one single band or two single bands of artificial fibers as a reinforcement met the conditions of the minimum threshold for flexural strength defined in PN-EN ISO 4049:2019-07, which specifies that materials intended for the restoration of occlusal surfaces and for other dental surfaces should achieve 80 and 50 MPa, respectively [34]. However, this standard does not specify the minimum thresholds that must be met by composite materials regarding other strength parameters, such as maximum bending force, deflection at failure and Young’s modulus. The assessment of a composite’s suitability for a particular application is determined by the flexural strength and the Young’s modulus, where the former parameter denotes the material’s capacity to carry a failure load and the latter parameter is responsible for the rigidity, hence their inclusion in the comparative analysis. The main reason for the creation and application of hybrid fibers was to design an FRC that would make use of the advantages of the individual components of the hybrid reinforcement to offset their disadvantages. Carbon fibers differ from aramid fibers in many regards [29,36,39,40].

In the present study, the highest results of the four tested strength parameters among the specimens reinforced with one single band of fibers were achieved by the aramid group. Specimens from the aramid group were characterized by the maximum bending force of 63 N, which was three times higher than the value for the control group. The presence of a single band of aramid fibers caused a three-fold increase in deflection with a simultaneous increase in the Young’s modulus of over 140%. In the tests, the specimens reinforced with two single bands of aramid fibers proved to have the highest flexural strength. The reinforcement with two single bands of aramid fibers improved the flexural strength almost three-fold and the value of the maximum bending force increased by approx. 40 N. The lowest flexural strength with the lowest values of the maximum bending force was achieved by the specimens reinforced with two single bands of carbon fibers.

The diameter of the fibers applied for the purpose of reinforcement of composite materials has a significant impact on their bending capacity. The carbon fibers and aramid fibers applied in the tests met the predefined conditions regarding the optimum diameter of an elemental fiber, ranging from 10 to 16 μm [41,42]. Numerous authors have proposed varied percentage contents of fibers, and the values range from 0.5 to even 90 wt% [43,44,45]. Karacaer et al. [46] determined that the highest strength and wear resistance were achieved by dental composites with fiber contents ranging from 2.0 to 7.6 wt%. They proved that a high percentage content of artificial fibers applied as reinforcement in a composite material was not beneficial. A content above 7.6 wt% caused the formation of clusters of fibers that were not bonded with the matrix, which resulted in worse polymerization and, additionally, could weaken the FRC structure. In the present tests, specimens reinforced with one single band or two single bands of artificial fibers had contents of 2 wt% and 4 wt%, respectively. When comparing the results of the strength parameters of the specimens reinforced with a single band of fibers to the specimens reinforced with two single bands of fibers, it can be stated that the fiber content in the specimen affects all the tested strength parameters.

Dyer et al. [44] tested several variations of the orientation of the unidirectional bands of long fibers in composite materials and achieved results that confirmed that, in order to carry maximum loads, the fibers should be located in the tension zone (the bottom part of the specimen), which corresponds to the inner surface of the prosthetic restoration in the conditions of the oral cavity. Dos Santos et al. [45] in turn, suggest that the fiber band in an FRC should be placed in the middle of the specimen, which is in the vicinity of the neutral axis. Therefore, in the present tests, one single band of fibers was placed in the tension zone, while in the case of specimens reinforced with two single bands of fibers the first band was placed in the tension zone, while the second band of fibers was placed in the middle of the specimen in the vicinity of the neutral axis. It was proved that the second band located in the middle of the specimen did not always significantly improve the strength parameters. The presence of the second band of fibers significantly improved the flexural strength only in the case of specimens reinforced with two single bands of hybrid carbon–aramid fibers. It must be emphasized that the presence of the second band of carbon fibers in specimens reinforced with two single bands of carbon fibers significantly reduced the flexural strength, at the same time reducing the values of maximum bending force and deflection at failure. The results of the present tests confirm the results achieved by Shi and Fok [47]. For an FRC to have the highest flexural strength possible, the fiber band should be placed on the inner surface of the prosthetic restorations. This is a key location on the fiber band, which determines the greatest capacity to carry high occlusal loads occurring in the stomatognathic system [10,42,43,44].

Carbon fibers and aramid fibers are resistant to water corrosion, mold and rot. Their degradation in the oral cavity environment is minimal [48]. Therefore, it seems important to protect the FRC used in tooth restoration with a composite material to eliminate contact with saliva [9,23,30].

The bright yellow color of aramid fibers does not reduce the esthetic qualities of the composite materials, which was observed in the tests. Due to their black color, carbon fibers have an adverse effect on the color of the FRC, which discourages or even disqualifies the use of such fibers for the reinforcement of composite materials in dentistry. As proved by numerous scholars, the fiber color not only affects the esthetic qualities, but also influences many other factors, such as the depth of photopolymerization, degree of monomer conversion, shrinkage stress, microhardness and cytotoxicity [1,9,25,43]. Nan-Chieh et al. [49] proved that, in order to minimize the adverse impact of incomplete polymerization arising from the presence of fibers inside the composite, the polymerization time must be extended and, if possible, the polymerization must be performed repeatedly from different sides. Therefore, in the present tests, the polymerization time was extended from 24 s (recommended by the composite manufacturer) to 50 s for the purpose of complete curing of the specimens and polymerization of the resin located between the fibers. In addition, each specimen was subjected to polymerization from four sides and, in accordance with PN-EN ISO 4049:2019-07, the polymerization process was performed with the recommended sequence of exposures [34].

When analyzing the results of the microscopic observations, special attention must be drawn to mechanical degradations occurring as a result of the performed strength tests. It is worth observing that only brittle cracking was visible and no other mechanical degradations were observed in the control group. Numerous different types of failures were recorded in the test group. Therefore, it can be stated that the occurrence of mechanical degradations was directly related to the presence of artificial fibers as a reinforcement. Matrix imperfections initiated on the fiber–composite boundary were observed very often. They refer to phenomena that occur both inside and on the boundary between layers. Such types of damage in the form of cracking are usually initiated by an accumulation of scattered microcracks (debonding) [24,50]. From the point of view of FRC mechanics, the cracking of the boundary layer, caused by the loss of adhesion on the fiber-matrix contact border, is very important. This cracking occurs during the earliest stages of the composite degradation process. Usually, this phenomenon initially does not affect the macroscopic characteristics of the structure but it might cause successive changes in the structure that lead to the FRC failure.

Aramids belong to a group of synthetic fibers produced from polyamides, i.e., compounds containing a characteristic amide group –C(O)–NH– in the main chain of macromolecules. Rigid aramid macromolecules are oriented in the fibers parallel to the fiber axis, creating planes in which hydrogen bonds occur between macromolecules and strong covalent bonds along the main chain, which are oriented in parallel. Aramid fibers have high tensile strengths due to the arrangement of aromatic groups and amide bonds, creating adjacent, layered, ordered structures of high stiffness, a high orientation of macromolecules and their organization into three-dimensional spatial structures parallel to the fiber axis. In view of the above, it should be assumed that aramid fibers can form more bonds with the resin matrix compared to carbon or glass fibers [12,22,28,36,37,38,39,40]. The situation is slightly different in the case of carbon fibers. The carbon fibers used in the study consist of 90% carbon. Their structure can be described as a set of graphite-structured crystallites arranged in a more or less orderly manner along the fiber axis. The degree of graphitization results from the arrangement and ordering of graphene planes, which are considered to be the basic units in the carbon fiber structure. Graphene planes, overlapping one another, create crystallites. Important parameters from the point of view of the properties of carbon fibers are the distances between these planes, their orientation relative to the fiber axis and the degree of corrugation and finally the size of the crystallites. The arrangement of layers changes in the cross-section of the fiber [27,28,29,30].

As recommended by numerous researchers for the purpose of improving adhesion, the fiber surface was subjected to oxidation treatment by placing the fibers in hydrogen peroxide [51]. The aim was to achieve the activation of surfaces as a result of inclusion of active functional groups, such as hydroxyl –OH, carboxyl –COOH or urethan –CONH, which considerably improve the strength of the bond. The formation of a high number of weak bonds is of a greater importance for the improvement of adhesion than a low number of high-energy bonds and the manner of their distribution along the macroparticle. Following the completed process of the oxidation of fiber surfaces, a bonding system must be applied, bearing in mind that manufacturers of dental composites recommend that compatible bonding systems should be applied [52]. In the present tests, the G-Bond bonding system recommended by the manufacturer and compatible with the applied composite, Gradia Direct, was applied. Raszewski and Nowakowska [53] indicate that various types of bonding systems are often used for infiltration of fibers, sometimes in contradiction to the manufacturer’s recommendations. The cracking and rupture of fibers are regarded as the final state in the process of FRC degradation. The failure of fibers leads to the formation of a failure path, which runs through the local zones that were damaged previously, and, as a consequence, causes the loss of the structure’s capacity to carry loads due to the physical destruction of the structure [54]. When analyzing the images obtained as a result of the observations in SEM, one has to notice the different appearance of the ruptured aramid fibers compared to the destroyed carbon fibers. Carbon fibers cracked transversely and the shape of the cross-section of a single fiber was round or oval. The destroyed aramid fibers had a completely different appearance. During the microscopic structural analysis of the ruptured aramid fibers, longitudinal cracks were noticed in addition to transversal cracks. The obtained images prove that a single aramid fiber was tensioned under the influence of the applied force with simultaneous longitudinal cracking until a critical length was reached, and at this moment the fiber ruptured. The fiber degradation, which consists of the occurrence of transversal and longitudinal cracks, was characteristic only for aramid fibers. This characteristic type of failure can be explained by the completely different chemical structure and the special production process of aramid fibers in comparison to glass fibers and carbon fibers.

The analysis of the results obtained in the study led to the conclusions that aramid fibers used in both single and double strands can be recommended as reinforcements for composites due to their strength parameters. Moreover, the use of carbon fibers with a weight content of 4% in the composite material is not recommended because of the decrease in the flexural strength and reduction in the stiffness of the FRC material. Additionally, the boundary of the fiber–composite interface was the weakest point in the layers formed by the fiber-reinforced composite materials. As the number of layers of FRC materials increases, the likelihood of cracks running through the entire thickness of the sample and the appearance of delamination increases.

To summarize the performed tests, the incorporation of carbon, aramid and hybrid carbon-aramid fibers into organic matrix has a significant impact on the values of mechanical parameters of dental composites. The results indicate that particular attention should be paid to aramid fibers, which have been rarely used in dentistry so far. The results obtained in laboratory tests are promising, and further pre-clinical and clinical tests with the use of aramid fibers might prove to be very significant and lead to the replacement of the commonly applied glass fibers with aramid ones. Future research on FRCs should focus, among others things, on the improvement of adhesion of fibers with the composite material, because, as proven above, the fiber–matrix boundary is the weakest point in the whole system formed by layered composites.

Many different directions for further research can be considered. The mechanical strength tests performed so far should be extended to include, for example, compressive strength, tensile strength and aging resistance. The tests performed are part of the preliminary stages of planned in vitro tests. It seems that laboratory tests are of particular importance in cases where there is no actual possibility of conducting analogous tests in intraoral conditions. The large number of variables defining the mechanical properties of the tested material means that the obtained results may differ depending on the center that conducted the tests. As noted by Karbhari et al. [41], the resulting discrepancies may occur even if the same type of fiber is used in the tests.

## Figures and Tables

**Table 1:** Comparison of composition and parameters of the carbon and aramid fibers used.

Fiber	Carbon Fiber	Aramid Fiber
Manufacturer	TORAY 3K	Kevlar DuPont
Composition, wt %	C (99 wt%), other (1 wt%)	Poly(*p*-phenylene terephthalamide)
Basis weight *, g/m^2^	240	200
Diameter of elementary fiber, µm	≈10	≈15
Roving linear mass, tex	240	200
Poisson number	0.31	0.36

* the mass of the textile material per unit area.

## Data Availability

Data are contained within the article.
